# A resampling-based meta-analysis for detection of differential gene expression in breast cancer

**DOI:** 10.1186/1471-2407-8-396

**Published:** 2008-12-30

**Authors:** Bala Gur-Dedeoglu, Ozlen Konu, Serkan Kir, Ahmet Rasit Ozturk, Betul Bozkurt, Gulusan Ergul, Isik G Yulug

**Affiliations:** 1 Department of Molecular Biology and Genetics, Faculty of Science, Bilkent University, TR-06800, Ankara, Turkey; 2 Department of General Surgery, Ankara Numune Research and Teaching Hospital, TR-06100, Ankara, Turkey; 3 Department of Pathology, Ankara Numune Research and Teaching Hospital, TR-06100, Ankara, Turkey

## Abstract

**Background:**

Accuracy in the diagnosis of breast cancer and classification of cancer subtypes has improved over the years with the development of well-established immunohistopathological criteria. More recently, diagnostic gene-sets at the mRNA expression level have been tested as better predictors of disease state. However, breast cancer is heterogeneous in nature; thus extraction of differentially expressed gene-sets that stably distinguish normal tissue from various pathologies poses challenges. Meta-analysis of high-throughput expression data using a collection of statistical methodologies leads to the identification of robust tumor gene expression signatures.

**Methods:**

A resampling-based meta-analysis strategy, which involves the use of resampling and application of distribution statistics in combination to assess the degree of significance in differential expression between sample classes, was developed. Two independent microarray datasets that contain normal breast, invasive ductal carcinoma (IDC), and invasive lobular carcinoma (ILC) samples were used for the meta-analysis. Expression of the genes, selected from the gene list for classification of normal breast samples and breast tumors encompassing both the ILC and IDC subtypes were tested on 10 independent primary IDC samples and matched non-tumor controls by real-time qRT-PCR. Other existing breast cancer microarray datasets were used in support of the resampling-based meta-analysis.

**Results:**

The two independent microarray studies were found to be comparable, although differing in their experimental methodologies (Pearson correlation coefficient, R = 0.9389 and R = 0.8465 for ductal and lobular samples, respectively). The resampling-based meta-analysis has led to the identification of a highly stable set of genes for classification of normal breast samples and breast tumors encompassing both the ILC and IDC subtypes. The expression results of the selected genes obtained through real-time qRT-PCR supported the meta-analysis results.

**Conclusion:**

The proposed meta-analysis approach has the ability to detect a set of differentially expressed genes with the least amount of within-group variability, thus providing highly stable gene lists for class prediction. Increased statistical power and stringent filtering criteria used in the present study also make identification of novel candidate genes possible and may provide further insight to improve our understanding of breast cancer development.

## Background

Microarray studies aiming to identify differentially expressed as well as co-regulated gene sets and signaling pathways involved in different cellular states have greatly improved our understanding of breast cancer at the molecular level. The power of expression profiling using cDNA or DNA microarrays for distinguishing subgroups of breast cancers has been demonstrated by several groups [1-4].

The identification of an intrinsic gene-set exhibiting high variability among different tumor clusters has been informative in describing different subtypes of breast cancer samples. However, only a few papers have been published on gene expression profiles of normal cell populations in breast tissue [5-9]. Therefore, it is of paramount importance for the research community in the field of tumor biology to have access to gene lists that exhibit low variability in expression among tumors and yet are distinguishable from a normal tissue profile.

Meta-analysis of microarray datasets has the potential to lead to more comprehensive measures of the existing differential gene expression data and can therefore provide gene sets with a high diagnostic value. Meta-analysis of independent microarray datasets generated with the common objective of identifying differentially expressed genes in a certain type of cancer has also been performed for breast cancer. In a very recent meta-analysis study, Smith *et al*. identified differentially expressed genes between ER+ and ER- breast tumors by gathering 9 independent breast cancer microarray studies [10]. Another study used the power of meta-analysis to find out the relation of expression patterns of gene and chromosomal positions. More than 1200 breast tumors were collected from eight independent breast studies and candidate metastasis suppressor and promoting genes were found from a given set of chromosomal regions [11]. Similarly, Hu *et al*. were able to identify a new intrinsic gene-set for breast cancer subtype prediction by combining multiple microarray datasets to assess prognosis [12].

Several different meta-analysis approaches exist in the literature. In some, each individual study contributes rather independently to the meta-analysis [13-15] whereas in others the values are treated as members of a single study thus requiring a generalized normalization step [16,17]. Direct comparison of gene expression values from multiple studies may be relatively more problematic than comparing the effect size obtained from individual studies. Yet, analysis of combined raw data is beneficial when sample sizes of individual studies are small. Another important concern in meta-analysis is the determination of the minimum number of samples required to obtain statistically reliable results [18]. One possible solution to this problem is resampling; for example, one can use a *delete-d-jacknife *procedure in which a subset of data is excluded to find out the frequency of selecting a particular gene as differentially expressed [18]. The number of replicates required for producing stable differentially expressed gene lists could also be determined based on a related method known as *leave-one-out *resampling [19].

Existing meta-analytic approaches applied to different types of cancer show the power of a combined study for identifying novel genes not present in the existing literature (e.g., liver cancers) [20,21]. Invasive ductal carcinoma (IDC) and invasive lobular carcinoma (ILC) make up to 95% of all breast tumors (IDC: 50–80% and ILC: 10–15%) [2,22-24]. Although recent studies suggest differences between the expression profiles of IDC and ILC, the clinical progress, therapeutic response, and molecular signature, there are also many similarities between IDC and ILC tumors distinguishing them from normal breast tissue [2,5,23,25]. However, meta-analysis of gene expression differences between normal breast tissue and such a generalized set of breast tumors has not been reported to date.

In the present study, we primarily aimed to develop a novel methodology for the meta-analysis of independent microarray datasets. Using this methodology, we provide gene lists that (a) are discriminative of breast cancer types (IDC, ILC) and normal breast cell populations, (b) may yield breast tumor markers that are invariably expressed across independent experiments, and (c) provide a set of consistently differentially expressed gene candidates with potential discriminative ability for tumor subtypes. Using a method similar to *delete-d-jacknife*, a series of *d *sample size values have been tested to assess the extent of variability across the tumor samples and the stability of differential expression. Comparison of probability value distributions obtained for the test and randomized samples has led to determination of the degree of differential expression between groups tested. Accordingly, we report that the Sorlie *et al*. [1] and Zhao *et al*. [2] datasets were highly comparable. Our resampling-based meta-analysis led to the identification of genes not differentially expressed when analyzed independently. Predictive ability of the meta-gene set was independently supported in three other breast cancer microarray studies with information on breast normal and tumor tissues [5,7,8] using BRB-TOOLS [26]. A subset of the meta-gene-list was also used as a classifier to accurately predict different molecular subtypes, such as luminal/basal and ER+/ER- based on microarray datasets in which patient subtype classification was available [7,8]. Moreover, selected candidates from stable gene sets obtained from the meta-analysis were validated by real-time qRT PCR. Use of resampling-based meta-analysis combined with class prediction via available microarray datasets pointed to the existence of a tumor-specific differentially expressed gene-set with predictive potential for tumor subtype classification.

## Methods

### Data retrieval for resampling-based meta-analysis

Two independent microarray gene expression data sets, Sorlie *et al*. [1] and Zhao *et al*. [2], were downloaded from the Stanford Microarray Database (SMD); [27]. Gene filtering options of SMD were used for log transformation and median centering the data arraywise. Expression values that were missing in more than 20% of the data were excluded from the analysis. Details of tumor specimen histology, available on SMD, were used to restructure the experiments according to breast tumor subtypes as invasive ductal carcinoma (IDC), invasive lobular carcinoma (ILC) and normal samples. Datasets were combined with respect to probe IDs using a set of customized perl routines (source codes are available upon request). These two data sets combined resulted in an initial list of 4769 IMAGE clones (3465 unique genes) common in both datasets (see Additional file 1; Zhao dataset and Additional file 2; Sorlie dataset). A total of 139 IDC (38 samples Zhao, 101 samples Sorlie datasets), 29 (21 samples Zhao, 8 samples Sorlie datasets) ILC and 7 (3 samples Zhao, 4 samples Sorlie datasets) normal samples were available for further analysis.

### Data Filtering

Data were filtered separately for ductal and lobular samples. IMAGE clones with more than 50% missing data in either of the Sorlie or Zhao datasets were excluded from the common clone set. Data filtering was further improved by performing two-tailed Student's t-tests with equal variance (Matlab^®^) between the Sorlie and Zhao datasets for the IDC and ILC samples separately. Those clones with probability values less than 0.05 (after Bonferroni correction) were excluded from further analysis. This two-step data filtering resulted in a common set of 1726 IMAGE clones for the analysis of ductal and normal samples, and 2029 IMAGE clones for the analysis of lobular and normal samples. Upon taking the intersection of the ductal-normal and lobular-normal clone sets, 1522 IMAGE clones were available for the ductal-lobular analysis. The resulting clone subsets were further filtered by removing IMAGE clones with more than 40% missing data for the two groups in comparison (e.g., ductal and normal) in the combined data before application of the resampling steps. In addition, if an IMAGE clone had a sample size (of normal samples) less than the resampling sample size, data on this IMAGE clone was also removed.

### Resampling and statistical analysis

**Figure 1:**
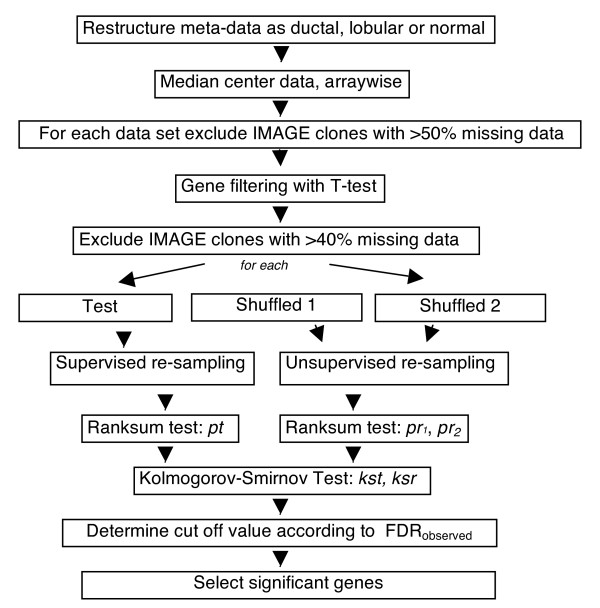
**General meta-analysis scheme**. Workflow is represented by boxes and arrows.

We have used a resampling method for meta-analysis of microarray data in which the significance of the difference between group medians (e.g. ductal vs. lobular) could be tested upon a series of resampling schemes from the original and multiple randomly shuffled datasets (Figure 1; code written in Matlab^® ^using Statistics Toolbox is available upon request). Accordingly, a preset number of samples was selected from each group (i.e., IDC, ILC, normal) of the original dataset, referred herein as the *test*. The p-value was calculated indicating the significance of the difference between the group medians based on the Wilcoxon Rank Sum Test. This test was repeated for a series of *i *number of iterations; at the end of each iteration scheme, a set of *p-values (pt) *per IMAGE clone was obtained. The above procedure was also applied to each of the shuffled datasets yielding *pr1 *and *pr2*. P-value distributions were then tested in a pair-wise fashion (i.e., *pt *vs. *pr1*; and *pr1 *vs. *pr2*) using the two-sample Kolmogorov-Smirnov test for each clone in the dataset (Figure 1). The resulting p-values were named as *kst *and *ksr*, respectively. To obtain an estimate of the false discovery rate (FDR), *ksr *values were sorted in the ascending order and the *k*^*th *^value from the top (lowest p-value) was determined as FDR_observed_, where *k *equals the expected value of FDR (e.g., 0.01) multiplied by the number of IMAGE clones tested. FDR_observed _was set as the threshold according to which IMAGE clones were assigned as significant or not. If *kst *of a particular gene had a value that was smaller than the FDR_observed_, the gene was accepted to be significant.

### Application to the breast cancer datasets

The above tasks were performed for a particular sample size *n *(e.g., 3), repetitively for *i *number of times where *i *= 10, 20, 30, ..., 100 and 150. For each particular *i*, three parameters were recorded, namely, *kst *values, the mean expression value of each of the two groups compared, and the significance of the differential expression based on *kst *and *ksr*. These above steps were then repeated with different sample sizes: For ductal vs. lobular comparison, *n *was set to be 3, 4, 5, 6, 10, 15 and 20. On the other hand, since the total number of normal samples was 7, the highest sampling value could be set to 6 for ductal vs. normal and lobular vs. normal comparisons, and *n *equaled 3, 4, 5 and 6. These sample size-iteration combinations led to 77 runs for ductal vs. lobular analysis, and 44 runs for ductal vs. normal and lobular vs. normal analyses. At the end, a final differentially expressed gene set was determined for each of the three comparisons (i.e., ductal vs. lobular, DL; ductal vs. normal, DN; lobular vs. normal, LN) by gathering the IMAGE clones that were assigned as significant in 90% or more of these 44 or 77 runs. The mean values of each of the two groups in comparison obtained at n = 20 (or 6, in the case of normal vs. tumor comparisons) and *i *= 150 were used as an estimate of the measure of expression.

### Data retrieval and analysis for validation studies

The ".cel files" of the three publicly available independent microarray gene expression data sets, GDS2635 [5], GDS2250 [7] and GDS1329 [4], were downloaded from GEO [28] and processed by the BRB-ARRAYTOOLS [26]. All three datasets were obtained using the Affymetrix HGU133A or HGU133 Plus 2.0 platform; thus they were highly comparable. In GDS2635 the aim was to identify gene expression profiles of microdissected ductal and lobular carcinomas in relation to their normal ductal and lobular cells (n = 10). The authors identified multiple genes differentially expressed in comparisons between ductal and lobular tumor and normal cells [5]. In the GDS2250 study, a gene expression array-based analysis of three breast tumor subtypes, i.e., sporadic basal-like cancer (BLC), BRCA-associated breast cancer, and non-BLC, was performed. They used 47 human breast tumor cases to provide insight into the molecular pathogenesis of BLC and BRCA1-associated breast cancer and the contribution of X chromosome abnormalities to the pathogenesis of BLC [7]. In GDS1329, Farmer *et al*. performed an analysis of tumors from 49 breast cancer patients that were successfully classified into luminal and basal classes, and a novel molecular apocrine class. Apocrine tumors were estrogen receptor negative ER(-) and androgen receptor positive AR(+), while luminal tumors were ER(+) and AR(+), and basal tumors were ER(-) and AR(-). Details of the breast specimens (normal-tumor, non-basal like- basal like, basal-luminal and ER (+)/ER (-)) available from GEO database were used in the supervised class prediction with a binary tree algorithm [26]. The common genes between the re-analyzed microarray studies and the meta-gene-lists were combined with respect to gene symbols (perl source codes are available upon request).

### Clinical Samples

Primary tumor samples and matched non-tumor breast tissues were obtained from patients (n = 10) during surgery and immediately snap-frozen in liquid nitrogen and stored at -80°C until RNA extraction. The frozen tissue samples were sectioned and mounted on glass slides. The slides were stained with hematoxylin and eosin for histopathological examinations. Only those tumor samples with more than 90% of tumor cells and matched tissue pairs with normal histological examination were included in this study. These frozen tissues were cut into 5-μm-thick sections and used for RNA isolation and cDNA synthesis. All the tumor samples had been classified as infiltrating ductal carcinoma. The use of the tissue material in this project was approved by the Research Ethics Committee of Ankara Numune Research and Teaching Hospital and consents were obtained in accordance with the Helsinki Declaration.

### RNA extraction and cDNA synthesis

The frozen breast specimens were put into Trizol reagent (AppliChem, Darmstadt, Germany), disrupted with a homogenizer and total RNA was isolated according to the manufacturer's instructions. Genomic DNA contaminations were removed by on-column DNaseI treatment (Macharel Nagel, Duren, Germany). The concentration of the isolated RNA and the ratio of absorbance at 260 nm to 280 nm were measured with the NanoDrop ND-1000 spectrophotometer (NanoDrop Technologies, Montchanin, DE, USA) in triplicate.

First-strand cDNA was synthesized from 1 μg total RNA using oligo(dT) primers using Revert Aid First strand cDNA synthesis kit according to the manufacturer's instructions (Fermentas, MD, USA). The cDNA was diluted at a ratio of 1:5 before being used as a PCR template and stored at -20°C until further use.

### Real-Time quantitative RT-PCR

Real-time qRT-PCR analysis was performed using gene-specific primer pairs (Additional file 3). Real-time qRT-PCR was performed on the BioRad iCycler Instrument (BioRad Laboratories, Hercules, CA, USA). The amplification mixtures contained 1.0 μl of 1:5-diluted cDNA template, 6.25 μl SYBR Green PCR Master Mix Buffer, and 10 pmol of forward and reverse primers in a total volume of 12.5 μl. Cycling conditions were as follows: an initial incubation of 95°C for 5 min and then 45 cycles of 95°C for 30 s and 60°C for 30 s during which the fluorescence data were collected. To verify that the used primer pair produced only a single product, a dissociation protocol was added after thermocycling, determining dissociation of the PCR products from 55°C to 95°C. Tumor and matched normal samples were always analyzed in the same run to exclude between-run variations and each sample was studied in duplicate. A no-template control of nuclease-free water was included in each run. An initial set of randomly selected genes from the DN list was used for real-time qRT-PCR validation studies. RAD21, GSN, COX6C, MAF, SFRP1, SPTNB1, GSPT1, NME1, PTTG1 but not MAF were also present in the LN list. Furthermore, seven other genes with potential predictive power for tumor subtype classification were studied by real-time qRT-PCR. These genes included *FN1*, *ID4*, *EGFR*, *ADAMTS1*, *ATF3*, *IGFBP6*, and *PRNP*. The geometrical mean of *ACTB*, *TBP *and *SDHA1 *gene expression values were used as internal control for relative gene expression quantitation [29]. Primer sequences and accession numbers of these genes were given in Additional file 3. The mean expression values obtained in resampling meta-analysis runs were used as a measure for comparing with the fold-change results obtained from the real-time qRT-PCR validation studies; a Pearson correlation coefficient was also calculated (Matlab^®^).

## Results

### Correlation of Sorlie and Zhao Datasets

**Figure 2:**
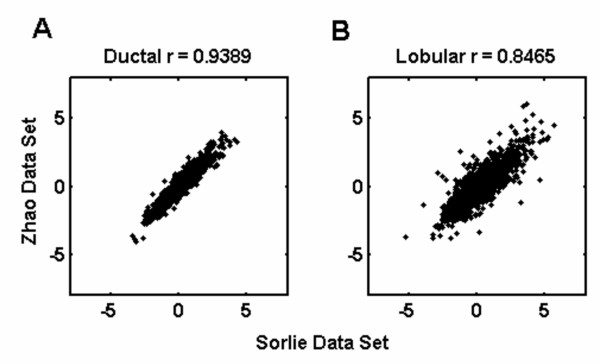
**Pearson correlation coefficients (r) between Sorlie and Zhao datasets**. Correlation plots between datasets after differentially expressed IMAGE clones were filtered out based on t-tests. (A) Correlation between mean expression values of ductal samples (p < 0.05). (B) Correlation between mean expression values of lobular samples (p < 0.05).

Combining the datasets in meta-analysis requires that they have similar expressions, both in magnitude and individual variability. To assess whether the Sorlie and Zhao datasets were correlated, a Pearson's correlation coefficient was calculated between the mean expression values of the ductal or lobular samples from each dataset, respectively before and after performing t-tests (Figure 2). Even before the removal of IMAGE clones showing significant differences between the studies, the mean expression values of ductal samples from Sorlie were highly correlated with those from Zhao; and a similar result was observed for the lobular samples (r = 0.8329 and 0.8233, respectively). After filtering out the differentially expressed IMAGE clones, the correlations between the aforementioned datasets increased to 0.9389 and 0.8465 for the ductal and lobular samples, respectively. These results ensured that there was significant correlation between the Sorlie and Zhao datasets although they were based on independent tumor and normal samples.

### Distribution statistics for generation of meta-lists

**Figure 3:**
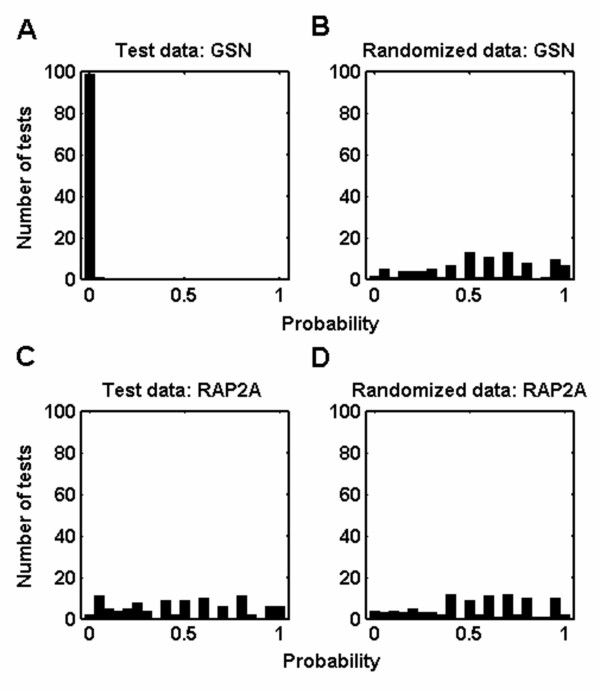
**Examples for probability distributions of Wilcoxon rank sum tests**. Data were obtained where resampling size, *n*, equaled to 6 (100 iterations). Assessment of significance was based on p-values obtained from the Kolmogorov-Smirnov test between test and random distributions (*pt *and *pr1*, respectively). (A, C) For test data, *GSN *gene had a highly significant differential expression (significant at 100% of iterations, p = 0.00) between ductal and normal samples whereas *RAP2A *gene did not (significant at 5% of iterations, p = 0.98). (B, D) Probability values of both *GSN *and *RAP2A*, obtained from randomized data, were uniformly distributed. *GSN*; IMAGE: 214990 and *RAP2A*; IMAGE:36684.

In this report, we used global-median normalized and filtered datasets since they minimized the number of manipulations performed during gathering of the meta-data (see Additional file 4). Accordingly, assessment of significance was based on p-values obtained from the Kolmogorov-Smirnov analysis between test and random distributions (*pt *and *pr1*, respectively) of a gene in the meta-data. For example, the *GSN *gene had a highly significant differential expression between ductal and normal samples as evidenced by the highly skewed distribution towards lower p-values whereas the *RAP2A *gene exhibited a uniform distribution of p-values (Figures 3A, B and 3C, D, respectively).

### Effects of resampling on estimates of expression and differentially expressed gene number

**Figure 4:**
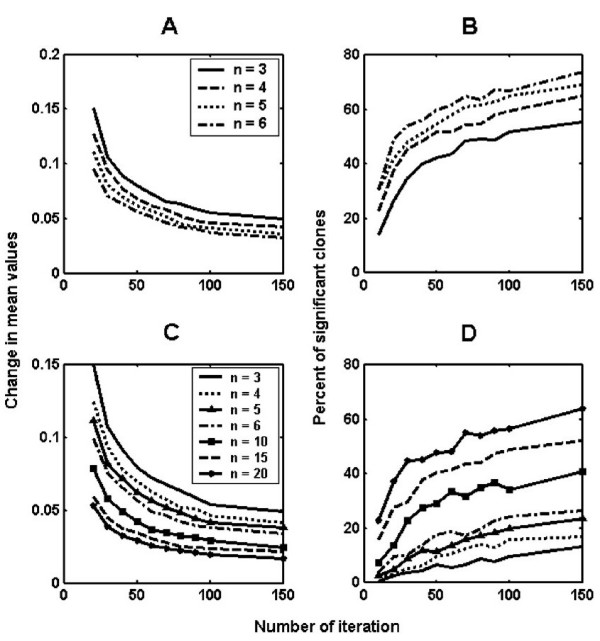
**Effect of change in sample size and number of iterations on mean expression values and number of significant IMAGE clones**. For each of the runs performed with different sample sizes (n), the change in the mean expression value (A, C) and the number of IMAGE clones that were stated as differentially expressed (B, D) were plotted with respect to the increasing number of iterations. A and B refer to the results of ductal vs. normal analysis whereas C and D show the results of ductal vs. lobular analysis.

We tested the effect of sample size and number of iterations on the estimation of mean expression level and the number of differentially expressed genes. For each run performed with a different sample size, the change in grand mean of expression (i.e., mean expression of all IMAGE clones) as well as the number of differentially expressed IMAGE clones were plotted with respect to the increasing number of iterations (Figure 4). As the number of iterations increased, the grand mean became more stabilized. Expectedly, the magnitude of change in mean values asymptotically decreased as the number of iteration and sampling size increased (Figure 4A and 4C). On the other hand, the number of genes stated as significant increased as a function of the number of iterations and sampling size (Figure 4B, 4D). Significant IMAGE clones made up more than 70% of all analyzed genes at sampling size 6 with the highest iteration in ductal vs. normal analysis whereas the same set-up resulted in only 20% significant IMAGE clones in ductal vs. lobular analysis.

It is reasonable to assume that use of a single sample size and iteration number may not be adequate to understand the variability among the tumor samples (Figure 4). It might instead be beneficial to consider all of the information gathered from the individual runs. Accordingly, the significant gene lists reported in this study were obtained by taking only those IMAGE clones that were assigned as significant in a given set of all resampling analyses performed (90% or more for ductal-normal, DN; and lobular-normal, LN; and 80% or more for ductal-lobular, DL comparisons) in an effort to minimize the effects of sampling size and iteration number on p-values.

### Characteristics of differentially expressed meta-gene lists

Differentially-expressed gene lists for DN and LN contained 298 (282 genes) and 216 (202 genes) IMAGE clones, respectively (see Additional files 5 and 6). On the other hand, there were only 66 (65 genes) differentially expressed IMAGE clones between the ductal and lobular (DL) datasets for 80% criteria (see Additional file 7). The size of these lists was dependent on the False Discovery Rate (FDR) input value (herein set to 0.01) or the percentage of resampling runs considered for significance (i.e., 90% or 80%). In order to obtain a larger number of genes for DL analysis, the significance percentage value was set to 80.

The same resampling procedures were also performed on the individual datasets, Sorlie and Zhao, separately. Compared to our meta-analysis these separate analyses together could provide 91% of IMAGE clones that were present in the significant DN list and LN list and 68% of the IMAGE clones of the DL list. However neither of the studies could supply 9% of the IMAGE clones of the DN and LN list and 32% of the DL list (90% cut-off), each of which corresponds to a novel contribution by our meta-analysis (see Additional file 8 for meta-analysis specific gene lists).

We also compared the final DL significant gene list with the list of 52 genes reported by Zhao *et al*. [2]. The DL list shared *CDH1*, *AOC3*, *FADS2*, *SORBS1*, *ALDH1A1*, *LPL*, *ANXA1 *and *AKR1C1 *with that of Zhao *et al*. [2]. However, our analysis did not assign reasonable significance to the F11 and VWF genes according to the set cut-off criteria (80%). The remaining genes in the Zhao gene list were not encountered since they were not included in the combined dataset used in the present meta-analysis. Meta-analysis of these two datasets provided a total of 36 significant genes not previously reported by Zhao *et al *and when either dataset is analyzed individually (see Additional file 7).

### Validation of tumor vs. normal meta-gene lists by independent microarray datasets

Recent meta-analysis studies identified common cancer signatures by combining microarray datasets from different tissues for increasing accuracy of tumor vs. normal class prediction [30,31]. In this study, we focused on extracting a stable tumor molecular signature based on two of the existing breast cancer studies that contain microarray data on normal, IDC, and ILC tissue samples. We also have validated the predictive power of the meta-gene lists obtained through the resampling-based meta-analysis using three additional breast cancer datasets, which contain microarray data on 3 or more samples of normal and tumor breast tissues (Table 1) [5,7,8]. Accordingly, subsets of genes from DN and LN meta-gene lists were able to predict the tumor vs. normal classes with high accuracies, ranging from 80 to 100% (Table 1). Strikingly, correlation between expression values obtained from significant discriminators from each of the three normal/tumor datasets and those from the meta-analysis was high (Table 1). This indicated that the DN and LN lists harbored a robust expression profile for the breast tumors when compared with normal breast tissue.

**Table 1:** Summary of GEO breast cancer microarray datasets and results of class prediction analysis for the meta-gene lists, DN (Ductal/Normal) and LN (Lobular/Normal).

Study GEO ID	Class		Meta gene-list					
			
			DN			LN		
	
	N	T	Accuracy (%)	Number of genes	r_DN_	Accuracy (%)	Number of genes	r_LN_
Turashvili [5] GDS2635	10	10	93	57	0.85	80	49	0.87

Richardson [7] GDS2250	7	40	100	145	0.86	100	96	0.78

Karnoub [8] GSE8977	15	7	95.5	109	0.72	95.5	89	0.81

Normal (N) and tumor (T) sample sizes, accuracy of prediction from binary tree algorithm (% accuracy), and the number of genes in classifier (number of genes) were shown for each study, separately. Correlation (rDN, rLN) of the classifier expression from each study with the DN and LN meta-gene expressions were also indicated (Pearson correlation, Minitab^®^; p < 0.001).

### Prediction of tumor-subtypes

We extracted a small, highly correlated classifier gene subset, which was commonly detected among the three microarray studies and the meta-analysis, to identify a more conservative gene set differentially expressed between tumor and normal cells (Additional file 9). Twenty-eight genes from the DN or LN meta-gene lists intersected with the three other microarray datasets (GDS2635, GDS2250, and GD1329); 17 of which were differentially expressed between basal vs. non-basal and/or ER status (Additional file 9). For example, *ADAMTS1*, *ATF3*, *IGFBP6*, *PRNP*, *EGFR*, *FN1*, *ID4*, *SPTBN1*, and *SFRP1 *genes from the DN list were found to significantly different in expression between nonbasal-like vs. basal-like tumors as well as basal and luminal subtypes of the breast tumors (p < 0.05). All of the above genes except FN1 were found to be significantly associated with the tumor ER status (p < 0.05; Additional file 9).

### Validation of ductal vs. lobular meta-gene list

Comparison of fold-change values of the DL meta-gene list consisting of 65 genes with that of the Turashvili's DL list (GDS2635) resulted in a high degree of correlation (r = 0.53; p < 0.001), suggesting that the direction and magnitude of expression change between the IDC and ILC samples were largely consistent between data from different microarray experiments. Furthermore, we combined published expression data from IDC and ILC samples from experiments performed by Bertucci *et al *[32] with the meta-analysis results (Additional File 7). Some of the members of the 65 meta-gene list were consistently down- or up-regulated also in the Turashvili and Bertucci datasets (i.e., down-regulated *ALDH1A1 *and *RBP4 *in IDC; and up-regulated *CDH1 *and *TFAP2A *in IDC). Protein expression levels of these four genes were investigated using the Human Protein Atlas, a public resource for immunohistochemistry (IH) of normal and pathological human tissues . IH data were available for CDH1, TFAP2A, and RBP4 proteins; and only data from antibodies exhibiting differential expression among breast tumors were reported herein. Accordingly, 2 out of 3 ILC samples exhibited moderate to strong signals for RBP4 (Antibody CAB00455) whereas 7 out of 9 IDC samples were either negative or had weak staining. CDH1 data in the Protein Atlas database was not very informative since the number of ILC samples were limited, but a moderate signal was detected for the ILC sample whereas 5 out of 6 IDC samples expressed CDH1 strongly (Antibody CAB000087). Similarly, TFAP2A was weakly or moderately expressed in the two ILC samples examined whereas a moderate to strong staining was observed in 5 of the 9 IDC samples. Although sample size in the ILC samples in the Human Protein Atlas database was limited, there was a corresponding trend between the mRNA levels reported by the present study and the protein level assessment obtained from the Human Protein Atlas. Future studies should include testing of the genes extracted by meta-analysis using protein level studies such as Western blotting or immunohistochemistry on a large set of IDC and ILC samples to confirm their predictive power.

### Validation of meta-analysis by real time qRT-PCR

**Figure 5:**
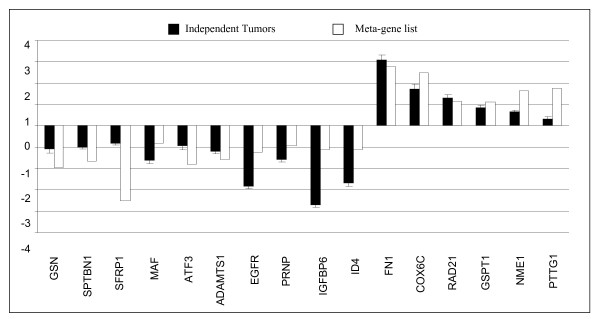
**Validation of meta-analysis results by real-time qRT-PCR**. Sixteen genes were selected from the ductal-normal (DN) significant meta-gene list for real-time qRT-PCR. Solid black bars refer to mean expression values (± SEM) of 10 independent IDC breast tumors normalized to their non-tumor pairs. White bars refer to the mean expression values from the combined meta-gene list.

We first selected nine genes that were found to be differentially expressed in both the DN and LN lists (except *MAF*) from the meta-gene list for validation of the meta-analysis. Expression profiles of these genes were tested in independent paired IDC breast tumor and non-tumor tissue samples through real time qRT-PCR. Our results were consistent with those of the meta-analysis such that *GSN*, *SPTBN1*, *SFRP1 *and *MAF *were down-regulated in most tumor samples with respect to their matched non-tumor samples whereas *COX6C*, *RAD21*, *GSPT1*, *NME1 *and *PTTG1 *were up-regulated (Figure 5). Additionally we selected seven other genes, *ATF3*, *ADAMTS1*, *EGFR*, *PRNP*, *IGFBP6*, *ID4 *and *FN1*, found to be differentially expressed according to tumor subtype and ER+/ER- classification from the tumor-specific differentially expressed gene-set. All except *FN1 *were found to be down-regulated in tumor samples with respect to their normal counterparts. The meta-analysis results were supported by the real-time qRT-PCR experiments since all tested genes exhibited differences between matched normal and tumor samples in the same direction as expected by the meta-analysis (Pearson correlation coefficient, r = 0.78, p = 0.001).

Among the genes we used for validation through real time qRT-PCR, *ID4 *was the gene found to be differentially expressed between DN only by meta-analysis rather than each study alone.

## Discussion

Microarrays allow high-throughput analysis of expression for thousands of genes and provide valuable information for tumor studies. For example, individual microarray studies have identified differentially expressed gene lists for distinguishing breast cancer subtypes and normal breast tissue [5,6,8,9]. Meta-analysis, on the other hand, might increase the knowledge by gathering and processing individual microarray datasets.

In the present study, we provided highly stable lists of differentially expressed genes based on meta-analysis of two breast cancer datasets [1,2]. We have used a resampling-based strategy in which the effects of number of iterations and sample size were minimized by using a voting scheme in which each IMAGE clone, at each run, was voted as either significantly- or non-differentially expressed and the significant counts then were added up. A percentage value was obtained by dividing the number of significant votes by the total number of votes and a threshold of 80–90% for each IMAGE clone was chosen as a cut-off value for this meta-analysis. The meta-analysis was able to report multiple genes (i.e., 29, 21, and 6 genes for DN, LN, and DL, respectively) which neither dataset could report when analyzed individually.

Sample size greatly influences the reproducibility of the significant gene lists, such that the lower the sample size the less stable the gene lists become [19]. In addition, Qui *et al*. [18] have shown that the stability of genes identified as differentially expressed varies: some genes are consistently stable whereas others are not, independent of the statistical methodology used. Along these considerations, our voting scheme provided an advantage for extracting highly stable gene lists.

Different statistical methods are available for assessing differential expression. Among these, non-parametric tests allow for comparison of low sample size and distribution-independent comparisons. Our choice of rank-sum test was based on this idea; similarly, previous studies reported the use of Kolmogorov-Smirnov test statistics to compare the reference and sample distributions in the context of Gene Ontologies [33]. We used a Kolmogorov-Smirnov test statistic for comparison of test and random distributions of p-values obtained from rank sum tests. In generating random datasets, we applied a gene-wise permutation algorithm that preserved the expression level information. Based on gene-wise permutations, a set of probability values that compare the actual and randomized distributions allowed for the assessment of the significance of the difference between groups tested using the Kolmogorov-Smirnov tests.

Different studies can be normalized and directly compared to each other in meta-analysis. Our comparisons ensured that there was a significant correlation between the Sorlie and Zhao datasets although these studies were based on independent tumor and normal samples; and the experimental procedures (e.g., amplification of RNA) also varied considerably between the two studies. Median rank scores [16] or quantile discretization algorithms have frequently been used to transform gene expression values from different studies to a common numerical range [17]. Since the global median-normalized and quantile-normalized data correlated well (see Additional file 4), we have used the former normalization method, with the least number of data manipulation steps, before combining these two datasets.

Due to the large number of comparisons involved in microarray data analysis, it is important to take into account the false positive error rate and control it for the number of tests performed. FDR is a well-known methodology for multiple-test correction; its estimation relies on calculation of the number of false positives in a randomly permuted set of experiments [34]. Therefore, we made comparisons between randomly shuffled datasets to obtain an estimate of FDR; and kept the value of FDR low (% 0.01) to reduce the number of false positives.

Invasive breast tumors comprise 18 different histological types [24], most of which are classified as invasive ductal carcinoma not otherwise specified (IDC NOS). ILC, on the other hand, makes about 10–15% of all breast tumors and it is histologically characterized by uniform tumor cells arranged in single-files or concentrically localized around ducts [35]. ILC exhibit heterogeneity just like IDC; and a high-grade aggressive form of ILC known as pleomorphic lobular carcinoma (PLC) exists [36]. Bertucci *et al*. [32] reported that IDC and ILC were histologically and genomically distinguishable from each other among the ER(+) grade II invasive breast tumors. Furthermore, ILC molecular subtypes were reported to include the typical and IDC-like ILCs, yet the *CDH1 *mutation and/or underexpression was common but not universal to ILCs in general [35]. Low-grade breast tumors were generally characterized by ER(+), PR(+) and with limited genomic aberrations whereas high grade tumors were generally ER(-) and PR(-) and had complex karyotypic changes. However, molecular differences among subtypes may not surpass the differences between any tumor cell and the normal since the degree of genomic stability in normal cells would be relatively higher.

The other three studies presenting data on ILC and IDC, Turashvili *et al*. [5], Sorlie *et al*. [1] and Zhao *et al*. [2] have used a more diverse selection of tumor samples. Although IDC and ILC have distinctive clinical and pathological characteristics and differ in their ER status and metastatic behaviors [22], meta-analysis of Zhao and Sorlie datasets indicated that a small number of genes distinguished between the expression profiles of IDC and ILC patients. On the other hand, the number of genes that were differentially expressed between normal and IDC or normal and ILC samples was much greater. Indeed, Turashvili *et al*. [5] has also reported only 28 genes that were significantly differentially expressed between IDC and ILC samples, which were extracted using laser-dissection, a more recent methodology allowing for precise collection of a given cell population. These findings suggest that the degree of molecular differences between IDC and ILC are indeed smaller than those between the tumor and normal classes.

Comparisons between the meta-analysis and the Turashvili and Bertucci studies pointed out to *CHD1*, *TFAP2A*, *RBP4*, and *ALDH1A1 *genes as commonly modulated. Indeed, *CDH1 *is one of the best-studied discriminators for ductal/lobular breast cancer specimens in the literature by immunohistochemistry and at the genomic level. In breast cancer, reduced *CDH1 *expression has been found in 50% of invasive ductal carcinomas, whereas *CDH1 *expression was almost always absent in infiltrating lobular carcinoma (ILC) [1,2,5,32,37,38]. *TFAP2A *was shown to be highly expressed in ductal tumor cells while normal cells expressed *TFAP2A *in the inner glandular cell layer [39]. On the other hand, nuclear TFAP2 expression was shown to be higher in lobular than ductal breast carcinomas [40]. There is no report on *RBP4 *in the literature in connection with ductal vs. lobular breast cancer distinction while ALDH1A1 protein levels were shown to exhibit differences among the ductal carcinoma patients [41]. The candidates identified in the meta-analysis then are likely to be discriminatory at the mRNA level rather than the protein level since protein localization and variability in intensity might make the ductal vs. lobular tissue discrimination less clear. Therefore, it is of paramount importance that future confirmatory studies include use of independent ILC and IDC samples for quantitative expression profiling of the selected candidate genes.

On the other hand, analyses of Sorlie, Zhao, and Turashvili data showed that tumor cells were remarkably distinct from their respective normals in their transcription profiles implicating that whatever the subtype structure underneath, most of the variability among samples was due to changes during tumorigenesis. Accordingly, the idea that genes discriminating tumor from normal in a stable manner also may have information on the state of the tumorigenesis is a valid one.

Breast tumor subtype classification remains a complicated issue due to difficulties associated with the presence of multiple interacting factors such as the presence or absence of node-filtration, ER-positivity, metastatic potential, different degrees of genomic instability, and tumor cell origin. For example, basal like cancers have distinct molecular expression profiles and histological differences when compared with the luminal type [42]. Nielsen *et al*. [43] have categorized basal like breast cancer tumors as having variable levels of expression of one of the three stem/basal markers, namely CK5/6, EGFR, and c-kit. Luminal cell markers, on the other hand, include CK8, CK18, CK19, mostly characteristic of glandular and/or lobular epithelial cells [44]. However, both the basal and luminar histochemical markers may exist simultaneously suggesting that breast cancer is rather a heterogeneous tissue [45]. It is also evident that tumors with a triple negative status (ER-, PR-, HER2-) are more likely to belong to the basal type [43,46]. In general, gene expression studies associated the basal-like breast tumors with high proliferative abilities and thus having a worse prognosis when compared with the luminal subtype of breast cancers [1,47]. Thus identification of genes best classifying breast cancer into intrinsic molecular subtypes like luminal, HER2+/ER- and basal-like also allows determination of risk factors and likely prognosis for the patients. The importance of identification of these different subtypes is that they differ in clinical outcome and molecular subtype signatures thus help predict clinical outcome and response to therapy.

Differentially expressed genes between tumor and normal states (DN and LN) also keep information about intrinsic subtypes. Accordingly, meta-analysis identified *ATF3*, *ADAMTS1*, *EGFR*, *PRNP*, *IGFBP6*, *ID4*, *SFRP1*, *SPTBN1*, and *FN1 *with ability to classify tumors into basal and luminal subclasses. Additionally most of them accurately differentiated ER(+) and ER(-) tumors (Additional file 9).

Among those genes, *ID4 *was found to be a novel tumor suppressor gene in normal human breast tissues and epigenetically silenced in breast cancer cell lines and primary breast tumors [48,49]. As supporting information for our data, de Candia et al. suggested that the expression of *ID4 *in the mammary duct epithelium may be regulated by estrogen depending on the differential expression pattern of ID4 in ER(+) and ER(-) breast tumors [50]. *SFRP1 *on the other hand is a frizzled-related protein that plays a role in a variety of cellular processes, including control of cell polarity, cell fate determination, and malignant transformation. In previous studies, loss of *SFRP1 *was found to be associated with cancer progression and poor prognosis in breast cancer [51,52]. EGFR is known to be a positive immunohistochemical marker for basal-like breast cancers and it was shown to accurately identify basal-like tumors from microarray data with potential therapeutic implications [53,54]. Activating transcription factor 3 (*ATF3*) is a member of the ATF/cyclic AMP response element-binding family of transcription factors. It was shown to enhance apoptosis in the untransformed mammary epithelial cells while protecting the aggressive cells and enhancing cell motility. Array analyses indicated that ATF3 upregulated the expression of several genes in the tumor necrosis factor pathway in the untransformed mammary epithelial cells. However, the expression of several genes implicated in tumor metastasis including fibronectin (*FN1*) was upregulated in aggressive cells. ATF3 was also shown to regulate the transcription of FN1, one of the genes obtained in the present study. *ATF3 *gene copy number was at least doubled in 80% of the breast tumors examined; protein levels also were elevated in close to 50% of these tumors [55].

Since the normal vs. tumor classification was strikingly distinct based on meta-analysis, and a gene-set with the capacity for breast cancer subtype classification, we further analyzed a set of normal matched tumors for selected genes from the meta-gene list using real-time qRT-PCR. The selected 16 significant genes were shown to have expression profiles similar to those found from the meta-analysis. Our findings also suggested that these genes could be used as predictors of tumor status regardless of the origin of the reference samples, i.e., a matched or pooled reference tissue. Since the number of samples used in qRT-PCR was relatively small, increasing the sample size may help generalize our results to a wider range of breast tumor samples.

There was a high level of correlation between fold changes obtained from the DL meta-genes and those from the Turashvili dataset, regardless of the different sample extraction methods used in each study (i.e., frozen sections and laser-dissection, respectively). The meta-gene list discriminating between ductal and lobular breast tumor samples at the mRNA level requires further confirmation at the protein level to better assess discriminatory power. Future validation studies might concentrate on whether meta-analysis specific genes also participate in prediction of level of prognosis and/or time to disease-free survival.

## Conclusion

In this study, meta-analysis of two independent comparable microarray data sets allowed us to provide genes that are able to discriminate IDC and ILC and normal mammary cells from the tumors that either study by itself was not able to identify. We also provided highly generalized and stable gene lists that could be used for prediction of tumor or normal status. The meta-gene list for tumor/normal comparison had a striking predictive ability based on comparisons made with three independent microarray datasets. The resampling approach proposed herein has the ability to detect a set of differentially expressed genes, with the least amount of within-group variability. This meta-analytic approach thus provides a method to combine two or more independent cancer data sets leading to the identification of differentially expressed gene sets for better understanding of cancer development and progression.

## Abbreviations

DL: Ductal and Lobular; DN: Ductal and Normal; FDR: False Discovery Rate; IDC: Invasive Ductal Carcinoma; ILC: Invasive Lobular Carcinoma; LN: Lobular and Normal; qRT-PCR: Quantitative Reverse Transcriptase Polymerase Chain Reaction; SMD: Stanford Microarray Database.

## Competing interests

The authors declare that they have no competing interests.

## Authors' contributions

IGY, BGD and OK originally conceptualized the meta-analysis method and application to breast cancer datasets, and wrote the manuscript. OK and SK developed the meta-analysis algorithm; SK contributed to manuscript writing. BGD and SK applied the algorithm to breast cancer datasets and generated figures. BDG performed all BRB-tool analyses and carried out expression studies. ARO and OK wrote algorithms for data extraction and compilation. BB carried out the surgical removal of the sample tissues and GE, the pathological assessment of the surgical tissue materials. IGY coordinated the study and participated in its design. All authors read and approved the final manuscript.

## Pre-publication history

The pre-publication history for this paper can be accessed here:



## Supplementary Material

Additional file 1 **Complete list of common IMAGE clones.** The data represents 4769 common IMAGE clones from combined Sorlie and Zhao datasets from the study of Zhao *et al*. [2]. Click here for file

Additional file 2 **Complete list of common IMAGE clones.** The data represents 4769 common IMAGE clones from combined Sorlie and Zhao datasets from the study of Sorlie *et al*. [1]. Click here for file

Additional file 3 **Genes used for real-time qRT-PCR analysis.** Gene names, accession numbers and gene specific primer pairs used for real-time qRT-PCR analysis of the selected genes. Click here for file

Additional file 4 **Correlation of global median and quantile normalized data.** The figure shows the correlation of global median and quantile normalized data of ductal (D) and normal (N) tissue samples. Click here for file

Additional file 5 **Gene set differentially expressed between ductal (D) and normal (N) breast tissue samples.** The data provided represents the list of 298 IMAGE clones differentially expressed between ductal (D) and normal (N) samples with 90% significance. Click here for file

Additional file 6 **Gene set differentially expressed between lobular (L) and normal (N) breast tissue samples.** The data provided represents the list of 216 IMAGE clones differentially expressed between lobular (L) and normal (N) samples with 90% significance. Click here for file

Additional file 7 **Gene set differentially expressed between ductal (D) and lobular (L) breast tissue samples.** The data provided represents the list of 66 IMAGE clones differentially expressed between ductal (D) and lobular (L) samples with 80% significance. Click here for file

Additional file 8 **List of meta-analysis specific genes.** The file includes the list of genes differentially expressed between DN, LN, and DL samples. Click here for file

Additional file 9 **Validation of meta-analysis gene lists by three independent microarray datasets.** The file represents the gene lists obtained by comparing the DN and LN meta-gene list to the gene expression profiles of normal and breast tumor tissues from three independent microarray datasets. Click here for file
