# Supplementary material for: A resampling-based meta-analysis for detection of differential gene expression in breast cancer
Source: BMC Cancer. 2008 Dec 30;8:396. doi: 10.1186/1471-2407-8-396 (PMC2631593; doi:10.1186/1471-2407-8-396)
Supplement: Additional file 3 — Genes used for real-time qRT-PCR analysis. Gene names, accession numbers and gene specific primer pairs used for real-time qRT-PCR analysis of the selected genes. [file 1471-2407-8-396-S3.pdf]

Additional file 3. The genes and gene specific primers used in this study

Gene Name	Gene symbol	Accession No/primer sequence [5'-3']	Amplicon Size (bp)
RAD21 homolog (S. pombe)	*RAD21*<br>Forward<br>Reverse	NM_006265<br>accaatgccaaccatgactg<br>cttcctcttcctcttggctt	114
Gelsolin	*GSN*<br>Forward<br>Reverse	NM_198252<br>ttcgagtcggccaccttcct<br>tctgcaccaccacctcgttg	108
Cytochrome c oxidase subunit Vic	*COX6C*<br>Forward<br>Reverse	NM_004374<br>tcaggaaggacgttggtgtt<br>gcacgaatgctacagccata	138
v-maf musculoaponeurotic fibrosarcoma oncogene homolog (avian)	*MAF*<br>Forward<br>Reverse	NM_005360<br>tggagtcggagaagaaccag<br>gcttccaaaatgtggcgtatT	228

Secreted frizzled-related protein 1	*SFRP1*<br>Forward<br>Reverse	NM_003012<br>ccgagatgcttaagtgtgac<br>ctcgctggcacagagatgtt	163
Spectrin, beta, non-erythrocytic 1	*SPTBN1*<br>Forward<br>Reverse	NM_003128<br>ggatcacagacctgtacact<br>tctcaagatggactctctgc	173
G1 to S phase transition 1	*GSPT1*<br>Forward<br>Reverse	NM_002094<br>cacctgtggaatcctctcaa<br>cctggctctgcttcacttat	157
Non-metastatic cells 1, protein (NM23A) expressed in	*NME1*<br>Forward<br>Reverse	NM_198175<br>tgtgagcgtaccttcattgc<br>aagaatggacggtccttcag	173
Pituitary tumor-transforming 1	*PTTG1*<br>Forward<br>Reverse	NM_004219<br>cctcagatgatgcctatcca<br>atcatgagaggcactccact	127

Fibronectin 1	*FN1*<br>Forward<br>Reverse	NM_212476.1<br>gcaagaggcaggctcagcaa<br>gcggacctacctaggcaatg	112
Inhibitor of DNA binding 4, dominant negative helix-loop-helix protein	*ID4*<br>Forward<br>Reverse	NM_001546.2<br>tcctgcagcacgttatcgac<br>ctctctagtgctcctggctc	263
Epidermal growth factor receptor (erythroblastic leukemia viral (v-erb b) oncogene homolog, avian)	*EGFR*<br>Forward<br>Reverse	NM_005228.3<br>gcaagaggcaggctcagcaa<br>gcggacctacctaggcaatg	227
A disintegrin-like and metalloprotease (reprolysin type) with thrombospondin type 1 motif, 1	*ADAMTS1*<br>Forward<br>Reverse	NM_006988.3<br>ggctgatgttggaactgtgt<br>acacgtggcctaattcatgg	104
Activating transcription factor 3	*ATF3*<br>Forward<br>Reverse	NM_001040619.1<br>gcactccgtcttctccttct<br>agaacaagcacctctgccac	191

Insulin-like growth factor binding protein 6	*IGFBP6*<br>Forward<br>Reverse	NM_002178.2<br>attgtgaccatcgaggcttc<br>aggagcttccattgccatct	132
Prion protein (p27-30)	*PRNP*<br>Forward<br>Reverse	NM_000311.3<br>gcgagcttctcctctcctca<br>gtgttccatcctccaggctt	140
Succinate dehydrogenase complex, subunit A, flavoprotein (Fp)	*SDHA*<br>Forward<br>Reverse	NM_004168<br>tgggaacaagagggcatctg<br>ccaccactgcatcaaattcatg	86
Beta-actin	*ACTB*<br>Forward<br>Reverse	NM_001101<br>ccaaccgcgagaagatgacc<br>ggagtccatcacgatgccag	124
TATA box binding protein	*TBP*<br>Forward<br>Reverse	NM_003194<br>tgcacaggagccaagagtgaa<br>cacatcacagctccccacca	132
