# Supplementary material for: A resampling-based meta-analysis for detection of differential gene expression in breast cancer
Source: BMC Cancer. 2008 Dec 30;8:396. doi: 10.1186/1471-2407-8-396 (PMC2631593; doi:10.1186/1471-2407-8-396)

Additional file 4: The correlation of global median and quantile normalized data of ductal (D) and normal (N) tissue samples.

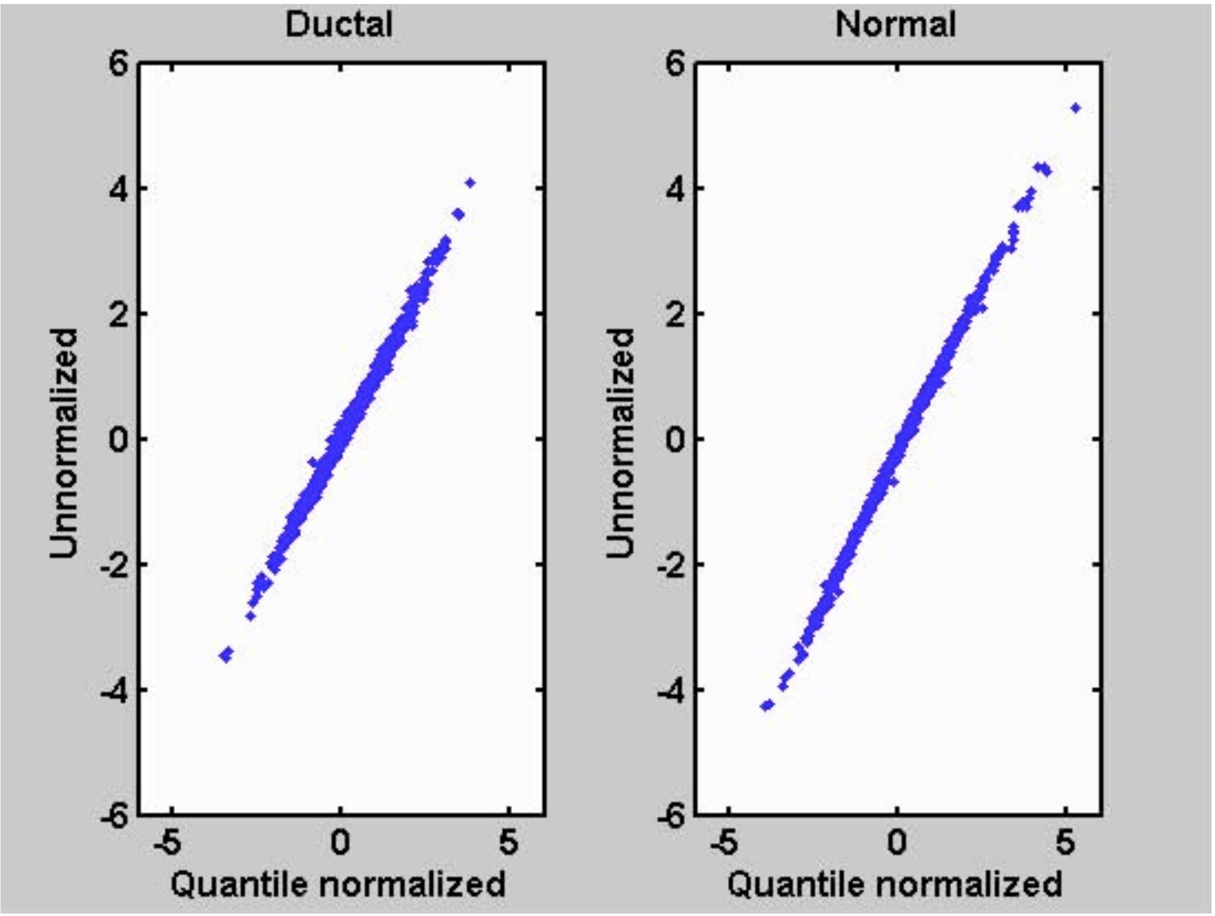

Supplement: Additional file 4 — Correlation of global median and quantile normalized data. The figure shows the correlation of global median and quantile normalized data of ductal (D) and normal (N) tissue samples. [file 1471-2407-8-396-S4.pdf]
